# Exploring the therapeutic alliance and race from sports psychologists' and athletes' lived experiences: A pilot study

**DOI:** 10.1016/j.heliyon.2022.e08736

**Published:** 2022-01-19

**Authors:** Jodine Williams, Ricardo G. Lugo, Andrea M. Firth

**Affiliations:** a Mind Advantage, UK; b Østfold University College, Department of Welfare, Management and Organisation, Norway; c University Campus of Football Business, Department of Psychology, UK

**Keywords:** Therapeutic alliance, Cross-racial dyads, Sport psychology, Cultural competence, Race

## Abstract

The therapeutic alliance has been explored widely within therapy literature but more research is necessary on the sport and exercise psychology client dyad. The racial/cultural identity development model and Rogers's (1957) six core conditions provide the conceptual and theoretical framework for this pilot qualitative study, which seeks to explore the building process in therapeutic relationships within cross-racial sport and exercise psychology. The perspectives of four black athletes of mixed descent and seven sport and exercise psychologists from diverse backgrounds (Arab = 1, Black British = 3, White British = 3) were considered. One-hour semi-structured interviews were analysed using an inductive thematic analysis. This yielded the following themes for athletes: lack of disclosure, racial impact on alliance, desired characteristics and experience. For sport and exercise psychologists these themes included therapeutic alliance building blocks, creating safe spaces and the racial impact of disclosure. Recommendations for building the process in cross-racial dyads are explored based on the participants' comments. The findings are discussed and areas for future research are explored based on these main themes.

## 1 Introduction

The quality of the therapeutic alliance, sometimes referred to as the working alliance between the psychologist and client, determines the efficacy of the change process (Orlinsky et al., 1994; Vasquez, 2007). Therefore, the relationship needs to be positive for necessary change to occur, and this relationship takes time to build. The therapeutic alliance requires effective rapport building to ensure the trust (Spiers and Wood, 2010) needed for effective change processes. The change process occurs when both the client and the therapist work together to achieve appropriate goals resulting in improved performance and/or well-being (Greenberg, 1986). Application of technique and personal attributes contribute to a positive alliance, allowing clients to feel understood and their issues appreciated when coming to therapy (Ackerman and Hilsenroth, 2003). Positive therapeutic outcomes are associated with the strength of the alliance between both psychologist and client (Duff and Bedi, 2010). Traditional research has explored the therapeutic alliance in therapy groups, but the subject of race within the athlete and practitioner therapeutic relationship has been neglected. Therefore, this research aims to explore building processes within the sport and exercise psychology working dyad.

The racial and cultural identity development model (RCIDM) (Atkinson et al., 1979) has been used to explore the cross-racial therapeutic alliance (West-Olatunji et al., 2007). This model conceptualises five stages through which an individual progresses when defining their racial identity. The first stage is the conformity stage: individuals have a preference for the dominant culture and may hold negative views of their own race. During this stage, both athlete and psychologist may prefer a therapeutic alliance from a member of the dominant race. The second stage is dissonance: occurring when identity is questioned, and individuals observe conflicting messages devaluating beliefs of their own culture, whilst simultaneously valuing that of mainstream culture. Such realisation elicits doubt in accepting mainstream culture and both athletes and psychologists at this stage may prefer a therapeutic alliance from a minority group. The third stage is resistance and immersion: rejection of the dominant culture occurs, and appreciation of their own group's attitudes is evident. Clients at this stage prefer sport psychologists of the same racial or ethnic group and may think their problems are a result of oppression. The fourth stage is the introspection stage: individuals begin to question rejection of mainstream culture whilst experiencing conflict with loyalty to their own racial group. At this stage the client prefer sport psychologists from their own racial group but are open to sport psychologists from another group if they have a similar world view. In the final integrative awareness stage, individuals can objectively accept or reject other group values. Individuals understand what they believe and can hear the values of others. Clients are open to a sport psychologist of any race, but prefer someone with similar world views and beliefs to their own. Crucially, in cross-racial alliances, understanding the view of the other (Turner, 2020) in relation to the stage of RCIDM within which either individual finds themselves, will result in nuances directly and indirectly affecting application of the core conditions (Rogers, 1957) necessary to build an effective alliance.

The six core conditions necessary to aid in change during client work are warmth, genuineness in the relationship, empathy, unconditional positive regard, psychological contact, the client's perception of the therapist and client incongruence (Rogers, 1957). A lack of adherence to these six conditions may result in ruptures in the therapeutic alliance, because the quality of the alliance is attributed to therapist actions and characteristics (Del Re et al., 2012). A lack of empathy can be one cause of ruptures, i.e. ongoing problems in establishing a therapeutic relationship or a negative shift in quality (Safran, 1993) within the client/psychologist relationship. This is especially pertinent where differences in cultural norms lead to a lack of empathy (Nelson and Baumgarte, 2004), causing a breakdown in the alliance, rendering the sessions ineffective. Poor understanding of the other, accompanied by a lack of compassion and emotional empathy during cross-racial working may result in ruptures, leading to disagreement of treatment goals and tension in the therapeutic alliance (Safran et al., 1990; Safran et al., 2011). This ultimately, in some cases, causes termination of the working alliance (Constantine, 2007).

Early session termination has also been linked to overt discrimination and microaggressions based on racial differences, which can lead to client discomfort and confusion (Roger-Sirin et al., 2015; Yeo and Torres-Harding, 2021) with 53% of racial and ethnic minority clients experiencing microaggressions within the therapeutic alliance (Owen et al., 2014). Such experiences and consequential terminations are more likely in African American clients (Constantine, 2007), who have criticised therapists who were culturally ignorant and insensitive (Thompson and Alexander, 2006). Conversely, those therapists displaying culturally specific knowledge, understanding of race and culture and how these shape individual life experiences were praised by clients. Strengthening of the alliance and cultural awareness are key in cross-racial working, and to help strengthen the therapeutic alliance practitioners need to understand the cultural meaning attached to the life events of clients and translate this into impactful practices (Day-Vines et al., 2007). The American Psychological Association (APA) (2019) and British Psychological Society (BPS) (2020) competencies state that being culturally skilled is necessary and counsellors should work to eradicate their prejudices. Despite clear guidelines from psychology governing bodies and recognition of the importance of properly addressing racial issues with clients, there is evidence therapists are not adhering to these recommendations (Roger-Sirin et al., 2015).

Traditionally, multicultural training has not occurred within the sport and exercise psychology educational pathway, although the BPS (2017) guidelines state an expectancy for all psychologist to work with various communities. It is expected that sport and exercise psychologists should be equipped with the necessary skills to work with people of different races, cultures, and religions, but no formal training occurs to enhance sport and exercise psychologists' skillsets. Skills needed to work with different cultures are not innate and require training, and this could aid effective alliance building in cross-racial work (Delgado et al., 2013). Due to the lack of diversity within the psychology field, matching clients with a sport and exercise psychologist of the same race or culture may not be possible. This can warrant the need for culturally competent practices within client work.

Wade and Bernstein (1991) researched the effect of cultural sensitivity training for counsellors and the effect of the counsellor's race on black female clients. The training was based on Pedersen's model of cross-cultural counselling (Pedersen, 1985) and covered the issues and concerns that different cultures may bring to training and counsellor self-awareness. It also highlighted how cultural sensitivity training improves cross-racial therapy and that counsellors who received such training received higher ratings by clients on empathy, trustworthiness and unconditional regard independent of their race. Thus indicating race plays a role in the therapeutic alliance and the nature of what is disclosed to therapists.

Working with diverse client groups involves interactions with various populations including different races, religions, and cultures, each with unique experiences that they bring to sport psychology sessions. A client's world view can be shaped by their perceptions and experiences, and can impact issues presented during sessions. For example, within some communities working with a sport and exercise psychologist carries negative connotations, which may lead to a lack of openness from clients. Additionally, a lack of cultural understanding by psychologists can lead to stereotyping and incorrect diagnosis (Roger-Sirin et al., 2015). Furthermore, ethnic minority clients are more likely to report differences in the therapist's ability to develop a working alliance and real relationship over time (Morales et al., 2018). Cultural competence, having the skill set to work effectively in any cross-cultural therapeutic relationship (Lo and Fung, 2003), is essential when working in multicultural communities (Sue et al., 2009). Cultural competence is explored widely within healthcare research and shows the need for better multicultural training and diversity of staff in the field, which will lead to better interactions with those from different backgrounds (Betancourt et al., 2016; Morales et al., 2018). However, caution is necessary, for cultural competence may lead to the stereotyping of clients resulting in inaccurate assumptions of the client and ineffective working alliances (Saha et al., 2008).

### 1.1 Importance of addressing race

Addressing race can be an important tool in building the therapeutic alliance. The field of psychology in the United States is largely dominated by white Americans, who make up 84% of the profession, while only 16% of practitioners belong to ethnic minorities (U.S Census Bureau, 2018). The BPS and HCPC in the United Kingdom do not provide such demographic information, resulting in a lack of clarity on the issue of race within therapeutic alliances in cross-racial dyads.

In order to address some of the potential issues within the therapeutic alliance, discussions of race at the onset of therapy can improve; client retention, the quality of the therapeutic alliance and treatment outcomes (Cardemi and Battle, 2003). Creating a safe space during cross-racial working between Caucasian and African American therapists allows freedom to discuss race issues during client work (Knox et al., 2003). Even so, there is still reluctance to discuss some racial topics, due to client mistrust of therapists and difficulty broaching racial topics. In order for therapy to work well race discussions should be initiated slowly and openly by the therapist (Hare, 2015). Discussions should include the impact of race on the therapeutic alliance allowing psychologists to actively include a multicultural element into their practice. Diverse approaches in discussing race varies from a series of conversations covering the possible impact race has on the client, the impact race has on the client therapy relationship, and the impact race has on the therapy process (Cardemil and Battle, 2003). These conversations will be different for everyone, as timing, appropriateness and pace are important, but these conversations can aid in building the therapeutic alliance. Furthermore, self-disclosure and sharing personal experiences allows for a strong therapeutic alliance and treatment satisfaction. Improving therapeutic alliances and the therapeutic bond by allowing both clients and therapists to address their honest views on race, leads to strong alliances and clients feeling understood (Burkard et al., 2006).

Stereo-typical racial connotation such as ‘Africans run pretty fast’ (Massao and Fasting, 2010) can lead to racial groups feeling isolated within sport (Cater-Francique et al., 2013), highlighting the potential presenting problems sport and exercise psychologists may face during sessions. Given the diverse multicultural clientele within sport, practitioner knowledge and understanding of different client backgrounds to help build effective cross racial/cross cultural alliances is vital. The APA states that the role of the sport and exercise psychologist has broadened from solely dealing with sport and exercise-related topics, and they are now dealing with a plethora of topics including, but not limited to abuse, mental health issues and helping athletes be advocates for a cause (Weir, 2018). This means topics of race may arise in cross-racial working, requiring sport and exercise psychologists to be skilled in addressing these topics.

### 1.2 Cross-racial working

The effectiveness of treatment is not always impacted by race. Race is sometimes not as important to the athlete. Instead, the qualities of the therapist take precedence (Horst et al., 2012). These qualities, as proposed by Rogers (1957), encompass; compassion, being supportive, good listening skills and competence. They allow the client to feel safe within the therapeutic environment in order to meet individual treatment goals, resulting in client satisfaction (Chang and Berk, 2009) irrespective of the need to address race issues.

The importance of cohesion within a working alliance is further highlighted in the supervisor—supervisee relationship. Supervisees felt topics of race were downplayed, avoided or minimised, and phrases such as “Let's not focus too much on the racial stuff” were highlighted by supervisees (Constantine, 2007), showcasing the mishandling of race issues within supervision, and gaps in current practice where appropriate training is needed.

### 1.3 Purpose of the study

Previous studies have shown the issues involved in developing and maintaining an effective therapeutic alliance in cross-racial therapy. Using a qualitative design, the rationale for the current pilot study was to gain a deep and better understanding of the experiences, of both client and practitioner, when building cross racial alliances within sport and exercise psychology. The study is a pilot for a larger mixed methods study both concerned with how comfortable athletes feel in expressing themselves and in raising racial issues with their sport and exercise psychologists. Further, the research sought to explore the experiences of sport and exercise psychologists' when working with athletes of a different race than their own, and how they dealt with racial issues if and when they arose. Delving into this issue, can allow for insight into cross-racial issues within the sport and exercise psychology field, highlighting the need for better race and cultural training.

## 2 Method

### 2.1 Research design

The study used thematic analysis of semi-structured interviews to deeply explore lived experiences involving the therapeutic relationship between athletes and sport and exercise psychologists during cross-racial sport psychology work.

### 2.2 Participants

Participants consisted of four competitive athletes (3 males and 1 female), of black British and black African descent, who were competing at a professional level in their sport. Two were track and field athletes, one basketball player and one footballer, the mean age was M: 25.3 (SD: 5.7). Participants also included seven practising sport and exercise psychologists (3 males and 4 females) from diverse backgrounds (3 Black British, 1 Arab, 3 White British). They had a minimum of two years' experience and an average of six years' experience between them.

The primary inclusion criteria for athletes was participation or previous participation in competitive sport, having had a minimum of two sport psychology sessions or currently having sport psychology sessions with a sport psychologist from a different race than their own. For the sport and exercise psychologists, registration with the BPS was required. The practitioner needed to be working, or had worked, in a cross-racial dyad and have been practising for a minimum of two years. All participants who did not fit these criteria were excluded.

Participants were initially recruited via email which was sent directly to athletes and coaches of sports clubs. Those athletes who expressed an interest but did not meet the criteria were asked to refer someone, and this yielded two participants. Potential participants were then sent an information sheet giving further details about the study via email and, if participation was agreed, a time for a Skype call was arranged.

### 2.3 Ethics

Following compliance with expected research ethical procedure, ethical approval was granted by the University Campus Football Business Research Ethics committee (**UCFBREC19W281**). All ethical procedures as outlined in the ethical submission were followed.

### 2.4 Procedure

An in-person pilot interview was conducted. As there were no major changes in the research schedule, the results of the pilot were included in the final data analysis. Following the pilot interview, to comply with necessary social distancing restrictions, due to the Covid-19 pandemic throughout the United Kingdom, all other interviews were conducted via Skype. All participants interviewed via Skype were emailed an informed consent sheet for their electronic signature ahead of the interview. Following the interview, a debrief form was emailed to summarise the study. Skype was used to record all video interviews, which were stored on a password protected computer. These were then transcribed. All identifying information was removed and pseudonyms were assigned to each participant.

### 2.5 Data collection

Data was collected via one hour recorded semi-structured interviews, with a core of twenty-three questions for both athletes and sport and exercise psychologists.

Despite the difficulty in building rapport using Skype interviews (Sullivan, 2012), the current research found that Skype interviews still allowed for the presentation of self in an authentic way. To prevent initial bias, both the researcher's and the participant's camera were turned off. Had the participant wished, they could choose to keep the camera off, but no participant chose to do so. Therefore, both the participants and researcher's cameras were on for the entire interview enabling visibility from the midsection upwards including the hands allowing the researcher to capture as much non-verbal behaviour as possible. No participants enquired about support for mental distress at any point before, during or after the interview process.

### 2.6 Data analysis

The six-step process as outlined by Braun and Clarke (2006) was used to conduct an inductive thematic analysis. In line with the six steps I read and reread the transcriptions to familiarise myself with the data, and initial impressions of the data were noted in a mind map. Initial impressions were formulated from thoughts that arose while rereading the data, and separated into athlete and sport and exercise psychologist groups. For example, respondent 1 explains “if I'm a different race than them, a different background, they might not feel like I'm able to relate to their struggles. Erm, but I would be more than happy to talk to them about it. For them to offload, vent their frustrations and emotions behind that.” As other sport and exercise psychology participants expressed a similar view, this led me to the initial impression “Understanding that racial differences can lead to a lack of disclosure and openness.” For triangulation, raw data was sent to the second author/supervisor prior to initial coding being conducted, and after initial codes were generated by the primary researcher these were discussed. Following this, the raw data was again sent to the second author for discussion of the themes created.

In step 2, initial codes were generated using a two-column table in Microsoft word, and quotes that linked to the research were highlighted for later usage. Using an iterative process, where data was analysed simultaneously to data collection data. The information gleaned through this process informed future data collection and was instrumental in ensuring saturation of data had been achieved.

For the third step, initial codes for each participant were entered in an Excel spreadsheet in columns and colour coded for each participant; athletes' and sport and exercise psychologists' codes were on separate tabs. Related verbatim quotes for each participant were grouped alongside initial codes to create initial themes.

The fourth step involved reviewing the themes to ensure they were clear and relevant to the research. For sport and exercise psychologists the themes were: therapeutic alliance building blocks, creating safe spaces and racial impact of disclosure. Themes for athletes were: lack of disclosure, racial impact on alliance, desired and experienced characteristics. This was done by reflecting on what each theme says about the data. For example, the theme ‘Lack of disclosure’ explains the phenomenon of lack of disclosure surrounding racial sporting experiences from athletes. Themes were then reviewed and refined by looking at all initial codes in Excel and grouping these. No codes could be placed under more than one theme and thus there were easily attributed to the relevant theme.

Thereafter, the themes were then defined and named in Excel. Related codes and quotes were grouped under each theme, and these were colour coded to match those of the participants.

For the final step, the themes were reported by looking at athletes and sport and exercise psychologists independently and comparing their responses for further discussion.

### 2.7 Reflexivity

The choice of research stemmed from interactions with personal clients of the same race (I am black British). These interactions led to an exploration of cross-racial therapy literature. Reflexivity allows for self-awareness and challenging of perceptions. As an athlete, after an injury, I experienced cross-racial sport psychology as a client which I found ineffective. This was due to lack of understanding and empathy from the therapist of my viewpoints, perceptions and personal experiences of the situation. These differences in understanding led to an eventual termination of therapy. To avoid my own experiences leading to bias, i.e. a distortion of results (Galdas, 2017), interview questions where reviewed by another researcher, of the same race prior to use. Likewise, to limit the occurrence of the falsification of the results, codes and themes were also reviewed by a second researcher.

To further avoid bias within the research and mindful that qualitative research has been criticised for lack transparency regarding how validity relates for qualitative methods (Flick, 2018), the research endeavoured to encompass the eight specific foci suggested by Tracy (2010). In that, the research identified a worthy topic, one which sought cultural understanding of the therapy experience. The research was conducted using *rich rigour* especially through the triangulation methods adopted, the research took pains to ensure *sincerity* both by the rapport built with each participant, data analysis, and the representation of the research findings. The sources used and the methods of data collection were *credible* and *resonance* is ensured by the evocative representation of the topic and findings of the participants lived experiences herein. The research is also *morally, practically and theoretically coherent* and was conducted using ethical procedures which paid attention the cultural sensitivity of the topic. Finally, the research strived towards *meaningful coherence* by utilising rigorous qualitative methods and appropriately interpreting the data in relation to existing theories and knowledge. To ensure validity in qualitative approaches, these eight aspects must be present at all stages to ensure epoché, or the process in not allowing one's biases and assumptions influencing phenomenological understanding and interpretation (Christiansen and Blumfield, 2010). This process happens at all stages of qualitative research: before data collection and in planning in therapeutic and interview sessions; during data collection by not influencing clients' responses and interactions; during data analysis by maintaining objectivity during the different steps of data encoding; and during presentation of the data in so to present results that is grounded in data and client experience.

The authenticity of my role in the research is based upon personal experience and belief in the importance for open and honest dialogue on racial issues within the therapeutic alliance (Burkard et al., 2006), especially when broached at an appropriate time during the relationship (Hare, 2015). Timing was a factor in the current research as there was some apprehension of how broaching the topic of race would be received in light of the current sensitivity surrounding Black Lives Matter (BLM) movement which, whilst originating in the United States of America, has taken on worldwide significance.

## 3 Results

The purpose of this research was to explore the building process within the sport and exercise psychology working dyad. Specifically, it explores the tools and characteristics that sport and exercise psychologists employ to build the therapeutic alliance and the qualities that athletes require during the process. The findings from the semi-structured interviews are presented individually and compared to show a crossover within the cross-racial dyad. For sport and exercise psychologists the themes were: therapeutic alliance building blocks, creating safe spaces, racial impact of disclosure and racial implications on the sport. Themes for athletes were: Lack of disclosure, Racial impact on the alliance, desired characteristics and experiences. These main themes will be explained using responses from the participants.

### 3.1 Athletes

#### 3.1.1 Desired and experienced characteristics

This theme captured the participants' views of the characteristics they desired their sport and exercise psychologists to have and those they experienced during their sessions. Participants showed an overlap with wanting to feel comfortable and understood. For example, Phoebe explains:

*"Erm I guess the main thing would be for them to like, for you to feel comfortable speaking to them and to sort of discuss anything you have on your mind or anything you want to discuss. I think the main thing should be like just feeling comfortable to actually just express what you want or just have space just to speak. "*

David also voiced the ideas of feeling comfortable and explaining that this can be done through an understanding of your client.

*"Erm firstly understanding, you know and trying to build a relationship as well and obviously I'd say that's a key one in terms of trying to work with any athlete. Once you can build a relationship with them and let them know that they have your understanding then they'll feel a bit more comfortable to let you know what is actually going on. You know, tell you about worries, anything stressing them, and I think they will be more open to listening to like what you have to say as well. So building a relationship and understanding and also them trying to put themselves in my position so they can understand what it is I am going through rather than just you know thinking, understanding and listening from their perspective and the position they're in now."*

David's explanation shows the importance of the therapeutic relationship for clients and how this will lead to their comfortability with the psychologist. Robert also discussed understanding as being a key quality:

*“I guess I want someone to understand where I'm from, you know, understand me as a black man or my culture a little bit to understand ok why are you having this situation. You know because I felt like some of the things.”*

Contrary to the above, Jacob explained that his main desired characteristic was the sporting background of the sport psychologist they work with.

*" I would prefer my psychologist to have erm been an athlete doesn't have to be the same sport but just be an athlete so yeah."*

When building a therapeutic alliance with a client, empathy and unconditional positive regard are key in aiding the change process (Rogers, 1957). These core conditions were described as understanding and safe spaces by clients. These athletes experienced that the characteristics did not match those they desired. Athletes were asked how their sport and exercise psychologists made them feel comfortable in sessions. Jacob and David explain the techniques their sport psychologists used to make them feel comfortable. Jacob explains that body language and tone were used to create a comfortable environment:

*“Erm… I would just things like, I don't know tone of voice, body language. erm things like eye contact really”*

Jacob then explained instances of empathy through understanding him and how this made him feel comfortable. He started:

*“Yeah I did because immediately as well he let me know that like when you're a foreign player going to other countries you're expected to carry the team and I think let alone a black foreign striker as well. Because he had been there for the club for a few years he's probably seen and spoken to other players. Some people buckle under it and have seen players come and go with expectations on them that's just ridiculous erm affected them as well. So he let me know from the get-go ‘I understand you're a foreign player, I understand that there's extra pressure on you,’ so that helped and that made our relationship quite positive”*

Empathy is a key factor in building the therapeutic alliance and encompasses understanding the client's needs (Rogers, 1957). David explains similar instances of how his sport and exercise psychologist got to understand him as a person and not just an athlete. He states:

*“Erm I would say at first he asked me things, everything else apart from sport like he wasn't trying to just focus on sport as a sense of like that's why I originally came to him anyway. He wanted to know about everything outside of sports, you know, before trying to find out what's going on or just trying to dive straight into the deep end and find out what was going on or why I came to him. He actually wanted to understand me as a person you know. General interests, hobbies and whatever. You know they make up life at the end of the day.”*

From his comments, the importance of understanding him as a whole person is seen and aligns with Jacob's experience of being understood. In contrast, Phoebe stated that she did not feel comfortable with her psychologist. She states:

*“Erm some umm somewhat but not really at the same time… erm yeah, I don't know I don't know if I felt completely comfortable”*

This does not align with her desired characteristics expressed earlier and could lead to a lack of rapport during her sessions. When desired characteristics were experienced during sessions athletes expressed an appreciation of this, as can be seen in David's comments.

### 3.2 Lack of disclosure

Three of the four athletes interviewed felt there was some information they would not share with their psychologist, whether that was about race or everyday life. For example, when asked if there were any racial issues they had experienced, they did not disclose these to their psychologists. Jacob states:

*" Ahh I mean every day. I think race especially there it's not like, it's not like England it's like a huge deal it's a regular thing. It's little jabs and it's joked off and you're expected to laugh it off; you can become very like a typical black guy who's just quick to get angry, who's aggressive. Also get seen as very aggressive if you react to a small racial joke, but it's still a racial joke. So yeah I think in terms of telling him it's like I didn't really tell him because it's, I don't, it's going to get shrugged off anyway. Because the manager would come out with racial jokes and the whole staff. I didn't need to tell him that, I already told people I speak to on the phone or family members, stuff like that really. "*

Phoebe reports similar examples of feeling that experiences may be ‘shrugged off’ or downplayed as she explains:

*"There was just like I remember being there and there was another girl there. She was another athlete she was doing rehab as well. Erm and she was just I just found her so annoying. She was just like really annoying and one day she must have said something which I found was quite like it was slightly racial insensitive and really just like ignorant. And I felt like she was being ignorant, and I must have pulled her up on it and said something to her about it. Erm so I guess it's something like that because I guess that's something that would impact my rehab process because I guess that's something that's on my mind. I wouldn't be comfortable saying that to any of the psychologists because it's just like I don't know if you might think the same way as her. You might just not understand where I'm coming from, you might just not think that it’s a big deal but to me it's a big deal."*

Phoebe's views support those experienced by supervisees when discussing race with their supervisors as they explain they felt their views were downplayed by those of a different race (Constantine, 2007). Clients feeling their views are being disregarded can lead to ruptures and withdrawal by clients (Chang and Yoon, 2011). Furthermore, Robert discusses there were perceived racial nuisances that could not be disclosed during sessions, as he believed this would not be received well.

*" Erm because black people are very combative in America so if I was to say that to somebody from my culture they can understand that because our ancestors come from situations you know. If I say that to you know this lady that is the wife of a gym owner. I don't want her to come home and tell her husband ahh man he wants to punch Thomas in the face and then the message gets spread around like. I'm smart enough to understand and I'm also that's how I felt, I didn't really want to hit him because that's bad for business but it was like truthful though. I don't want to smack and hit my coach in real life you know that's not good on anybody's resume you know but you know it's an expression."*

This highlights views that clients may feel they will be stereotyped if they express particular views that may be deemed normal within their race. Only one participant stated they would discuss racial issues with their sport psychologist:

*“I would say yes because it would be something that at the end of the day I would want to try and kind of just open up with someone”*

The idea that racial issues or racial expressions would not be discussed with a sport psychologist of a different race due to a lack of understanding was a central theme. As shown from research by Massao and Fasting (2010), racial issues are present in sport and this is supported by the participants' comments.

### 3.3 Racial impact on alliance and understanding

This theme captured thoughts on the perceived impact race had on the understanding of issues and the impact this would have on the alliance. When asked if they would disclose racial issues with their sport psychologist, all participants stated they would feel more comfortable discussing these experiences with a psychologist who is the same race as they are. For example, when asked if they would have discussed racial issues if their psychologist was the same race, David stated:

*“Erm maybe slightly as I would feel like he would probably have a better understanding than the current sports psychologist had. You know he would maybe have been able to relate to what I'd be saying or to what I would be trying to make him understand.”*

Although David said his sport psychologist made him feel comfortable, David would not broach the subject of race and how this may play a role in life and sports. As seen, race plays a role in some athletes' sporting careers and can impact performance. Jacob expressed the view that a black psychologist would have experienced racial jokes or microaggressions, which meant they would understand the impact for the client:

*“Yeah definitely because erm for example if you're black there's a high chance you've been through it so you would understand exactly what I'm talking about. You would understand that even a small racial joke, as much as I've got thick skin so it's nothing, it's still something.”*

The essential point made is that there is a shared understanding between people of the same race which allows them to understand how race impacts the everyday experiences in life. This was shown in research where Japanese clients preferred psychologists of their own background due to feeling more comfortable (Naoi et al., 2011). This highlights the importance of same race dyads and how they can lead to comfortability for clients. Similar views are held by Phoebe, who states:

“Yeah 100% I think I would be able to comfortably speak about that. Erm yeah because I think she would understand it more or he would understand it a bit better than they would.”

Robert stated:

“I think they can understand what it feels like, you know. If you have a person from the same race they can understand what it feels like to be in your shoes or be angry about something.”

The comments show that all athletes seek to be understood by their sport and exercise psychologist, and they feel this is better done with a psychologist of the same race. Even though all participants stated they would feel more comfortable expressing racial views with someone of the same race, they did not state they would only like to work with a sport psychologist of the same race.

### 3.4 Sports psychologists

#### 3.4.1 Therapeutic alliance building blocks

Sport and exercise psychologists explored their thoughts on the techniques they use to build a therapeutic relationship between them and clients. This section presents findings related to the qualities sports and exercise psychologists think are important for building therapeutic relationships, and how they implement these in their practice. Five out of the seven participants discussed empathy and authenticity as key qualities they bring to sessions. For example, Polly explains:

*“Empathy, I think for me I've always just been a very empathetic person. Erm I think the fact that I validate their feelings and kind of acknowledge that they yeah this is something that is, of course, is frustrating or this is something that yeah you're going to be sad about. And kind of validating.”*

The first concept identified by Rogers (1957) to aid building therapeutic alliance was empathy. This involves an understanding of clients presenting problems, and this was a core quality discussed by participants. Something similar was understood by Jane:

*“I think the qualities of kind of coaching and counselling, the sort of raw qualities of being a coach, counsellor or psychologist are empathy, compassion, combined with neutrality and challenge.”*

Empathy as a quality was expressed by Toby, whereas Sarah explains that creating a non-judgemental space is key:

*“Erm of course with all kinds of psychotherapy, an approach of sorts, of having that empathetic approach”*

Sarah explains:

*“I've realised the most important attributes that I certainly think you should have are non-judgemental caring and just being genuine.”*

Andrew states:

*“I suppose erm being authentic. So I think sometimes when you're working in sport. When you work as a psychologist there can be this idea, you can I suppose maybe try and be too professional and standoffish. I think that I tend to be like my regular self as often as I can, obviously abide by professional boundaries and ethics and stuff but barring that I just try and be myself.”*

Gregg explains similar techniques of being himself and showcases a genuine nature.

*“Probably be the way I can interact one-to-one with people and have relationships and building strong er relationships and allowing them to open up.”*

Empathy translated as a core point in the therapeutic building process is present in the current therapeutic literature. In addition, this is the first core condition presented by Rogers (1957).

### 3.5 Creating safe spaces

Five out of seven sport and exercise psychologists discussed how they create safe spaces for clients to express themselves. This arose from questioning regarding techniques used to make clients feel comfortable. For example, Toby states:

“*Umm I guess it just occurs naturally in the rapport-building elements, just politeness, empathy when they're sharing thoughts, letting them know as well even explicitly that there's no judgement within this, and to feel free to share anything*.”

This technique supports the six core steps presented by Rogers (1957), especially unconditional positive regard. This condition involves providing a safe environment for free expression from clients, where any topic can be discussed without fear of judgement. Polly discusses the environment she creates to allow free expression, as she states:

*“This is very much an open space, it's a chance for you to express those thoughts and feelings and I really do embrace that and say it's ok to express that… I'm very, very supportive of clients expressing themselves and you know using our session as a chance to really talk about things and erm yeah I'm very supportive of that.”*

Andrew's comments are similar, as he states:

*“I think it goes back to being authentic, I think if people can see that you are being yourself. I think that erm that helps people do the same. And also to start building psychological safety in the places that you're in, where people can experience that being themselves is not something that's going to come with negative consequences.”*

Andrew highlights another of the six core steps, which is congruence. This is the showcase of genuineness and authenticity, which allows for a trusting relationship to be built. Conversely, two of the sport psychologists talk about creating a physical safe space for clients, which is seen when Karen states:

*“I think you know it's just the usual tricks like you know I don't have a seat. I have two identical chairs, they can sit where they like, I never sit before they do. Erm I ask them if the lighting is ok because I can turn it up or down. I want them to feel comfortable”*

Similarly, Jane comments:

*“I think the main things I do are setting up the room and making sure the pre-work is there.”*

Participants' comments supported steps outlined by Rogers (1957), which include unconditional positive regard, empathy and congruence. These steps have been shown to foster a positive alliance between therapist and client (Del Re et al., 2012).

### 3.6 Racial impact on disclosure

Mixed comments were seen when discussing racial disclosure. This theme captured participants' thoughts surrounding whether race would impact the information their client shared with them. Four participants explained it might impact information shared, and three explained that it would not. For example, Sarah states:

*“Erm yeah, of course, I think it probably does. I wish it didn't but I think it probably does in a sense that if you, if they perceive you in a certain way they are potentially less likely to share certain aspects of their life.”*

The stages of the RCIDM (West-Olatunji et al., 2007) explain the attitudes clients may feel towards their psychologist if they are of another race. If clients are at the resistance and immersion stage a lack of disclosure may occur due to feelings of oppression by the dominant race. At this stage, these clients would prefer a psychologist from their own race, and the change process may be impacted if this does not occur. Similar to Sarah's views, Andrew explains:

*“It could because I think there might be a level of like, their expectancy about my ability to empathise with the situation. So if they didn't think I necessarily understand what it’s like to be in that situation or I couldn't relate to it then in those instances that would probably affect their willingness to want to share that with me.”*

These comments support research by Nelson and Baumgarte (2004) which shows a perceived lack of empathy by clients can hinder the therapeutic alliance, which can lead to ruptures, including termination. Both Sarah and Andrew are from minority backgrounds and highlight their belief that race may impact disclosure. Karen's points offer another perspective on racial experiences of the Caucasian population, which sheds light on the different racial views, as she states:

*“I mean with my black clients, because I'm black, I think if they were to talk about racial issues I don't get the feeling they would be uncomfortable doing that because you know we are both ethnic minorities erm the white people I don't really think they have any issues with race that I know of.”*

This is an interesting view, which shows her thoughts on the racial experiences of Caucasian people with sport psychologists from a different race. Furthermore, her comments explain her thoughts of same-race working, and the comfortability her black clients have with her. Contrary to these views, three other participants explain that they do not believe their clients would have trouble discussing racial issues with them. For example, Gregg states:

*“If I've set it erm the environment up where they felt comfortable to talk about it with me, which I hope I would have done, erm yeah I would like to think so.”*

These views are supported by research that explains feelings of comfortability and therapist skills are more vital than racial differences (Chang and Berk, 2009). Jane explains that there will always be differences with clients which can include sport, gender or other factors, as she states:

“No, I always feel like I've got obvious differences from the athletes I'm working with, because obviously I'm not them and I haven't done what they're doing.”

The athletes' comments in this study explain they currently face racial issues, which may highlight a lack of awareness from Polly of the racial issues faced in the current society. The views highlight the awareness shown by sport psychologists of the impact race can have on an athlete and their sport. Participants do not touch on the psychological factors that may occur due to racial issues, but are aware that they occur. Exploring responses separately has given insight into techniques for building therapeutic alliances, reservations with disclosure and qualities required for successful work. Across themes there are similarities between sport and exercise psychologists' comments and athletes' comments. For example, the qualities that sport and exercise psychologists outlined, such as empathy, were considered as needed by the athletes, which shows similarity in views surrounding this topic.

## 4 Discussion

The current study explored the building process within the cross-racial sport and exercise psychology working dyad and was informed by literature on cross-racial therapy work. Both athletes and sport and exercise psychologists reported their experiences in cross-racial work. Generally, athletes believed that understanding and feeling comfortable were necessary characteristics in the cross-racial relationship. They felt this was necessary to allow openness and ease of sharing their experiences. The characteristics brought to sessions outlined by sport and exercise psychologists include empathy, being non-judgement, safe spaces, and unconditional positive regard, which aligned with the views and requirements of the athletes and with those of Rogers (1957). Rogers's core considerations for therapeutic alliances state empathy as the first core principle in building a relationship conducive to change. It is fundamental to the therapeutic relationship because a lack of empathy has been shown to cause ruptures in the therapeutic alliance (Nelson and Baumgarte, 2004).

Although both athletes and sport and exercise psychologists had similar views on characteristics, in keeping with the findings of Yeo and Torres-Harding (2021) athletes explained there was information they would not share during cross-racial work. This was because they thought their experiences would be minimised, they would not feel comfortable, there would be a lack of understanding or empathy by their sport and exercise psychologists of their views on race and fear of how they would be viewed. When race or racial experiences are downplayed or minimised this can lead to a lack of cohesion and engagement from clients (Constantine, 2007). In addition, cultural mistrust between races (Townes et al., 2009) can lead to wanting a same-race dyad. The current study indicates that athletes felt their daily racial experiences in sport may be downplayed due to a lack of understanding. Thus, during cross-racial work clients may not divulges certain aspects of their negative lived experiences, if they feel their sport and exercise psychologist will not understand.

To aid in successful cross-racial work, addressing race early in the therapeutic process has been shown to improve the treatment outcomes and lead to positive therapeutic alliances (Cardemi and Battle, 2003; Morales et al., 2018). Sport and exercise psychologists in this study did not experience having to address race with clients and did not state they broached this subject. Unlike previous research that showed African American clients (Constantine, 2007) and racial ethnic minorities (Owen et al., 2014) were more likely to experience microaggressions, these athletes did not express the presence of this in their sessions. Contrary to athletes' feelings, all sport and exercise psychologists expressed they would be open to discussing issues of race with clients, but some understood why their clients may not want to disclose this information. Some participants said they understood that a perceived lack of understanding by athletes could lead to a lack of disclosure.

Whilst none of the athletes refused a sport and exercise psychologist of a different race, all athletes explained their preference for a sport psychologist of the same race as their own, because they felt this would help them be better understood. Similar results were shown with Japanese athletes and their preference for same-race dyads from sports consultants (Naoi et al., 2011). This supports the introspection stage of RCIDM, which explains that clients may prefer same-race working dyads, but do not reject cross-racial working ones. In comparison, the RCIDM did not align with the comments discussed by sport and exercise psychologists, as they were open to any race irrespective of their beliefs, values, or worldviews.

### 4.1 Implications for practice

This study highlights gaps in knowledge and training in the sport and exercise psychology field in relation to cross-racial working. The research has shown the six core considerations (Rogers, 1957) and cultural competence are effective in strengthening the alliance between clients and practitioners. The athletes in this study highlighted their preference for same-race working dyads, but if these are not available, providing an environment conducive to change is still important. As client's culture or race impacts their view of the world, life experiences and interactions (Burkard et al., 2006) it is important to be aware of these for better practice. Additionally, these athletes highlight characteristics that align with Rogers's (1957) six core considerations, which include understanding and feeling comfortable. Research has shown the importance of cultural knowledge for clients' satisfaction with sessions (Day-Vines et al., 2007) and this is echoed in this research when discussing lack of disclosure of racial issues. Athletes explained the perceived lack of understanding they thought their sport and exercise psychologist had in relation to racial or cultural issues, which hindered their disclosure.

Similar views are also prevalent in other current day dyads such as doctor-patient relations where minority clients still perceive and experience racial disparities in treatment (See Bedi, 2018; Sigurdson et al., 2019), therefore given its cultural prevalence, its occurrence in the sports therapeutic context is unsurprising.

Drustup (2020, p.182) addresses this problem:

*‘…the concept of whiteness, not as the existence of white skin, but as “the overt and subliminal socialization processes and practices, power structures, laws, privileges, and life experiences that favor the white racial group over all others” (inHelms, 2017, p. 718). Whiteness operates as a position of racial privilege, the lens through which people who are white see themselves and the world, and is a set of social and cultural practices that are often invisible to those in the dominant group (inFrankenberg, 1993).*

Beck (2019) outlines how therapists need to have the ability to establish the necessary rapport to ask about these experiences and be able to incorporate this information into therapy. Gibson (2020) wrote, “We all need to do the hard work, which involves critical reflections and changing the systems, structures, and processes” (p. 18, emphasis added).

Recent theoretical developments around Relationship-inferred Self-efficacy (RISE; Lent and Lopez, 2002) has highlighted how the alliance can be influenced by personal factors such as racial disparity. While therapists who are more similar to their clients are able to transfer knowledge and coping skills better, perceptions of distance between the client and therapist leads to worse outcomes.

With no formal cultural competence training present for sport and exercise psychologists, navigating these issues can be difficult. Cultural competence training within the sport and exercise psychology field could aid in cultural knowledge for practitioners and support them in the building process of cross-racial working dyads.

### 4.2 Limitations and future research

Although the current study explores the building process within the sport and exercise psychology working dyad, generalising these results to all black athletes or sport and exercise psychologists should be approached with caution. Firstly, although the sample size is similar to that of other qualitative research papers, this study only included four athletes, all of black descent. Inclusion criteria required athletes to have engaged in a minimum of two sessions with a sport and exercise psychologist of a different race than their own. This narrowed the scope of athletes able to participate and access to those higher-level athletes. This group might represent some athletes, but not all that have engaged in the cross-racial dyad. Despite limitations, this study provides support for the building process in cross-racial sport psychology dyads from both the athletes’ and the psychologists' viewpoints.

This research suggests valuable areas for exploration of future research. One area that would be of interest would be the comparison of cross-racial working dyads and same-racial working dyads in a sports setting, as participants discussed the comfortability with psychologists or clients who were of the same race as their own. Due to a lack of cultural competence training in the sport and exercise psychology educational pathway, research into the impact of cultural competence training on cross-racial sport psychology work would be relevant.

## Declarations

### Author contribution statement

Jodine Williams: Conceived and designed the experiments; Performed the experiments; Analyzed and interpreted the data; Contributed reagents, materials, analysis tools or data; Wrote the paper.

Ricardo G. Lugo, Andrea Firth: Contributed reagents, materials, analysis tools or data; Analyzed and interpreted the data; Wrote the paper.

### Funding statement

This research did not receive any specific grant from funding agencies in the public, commercial, or not-for-profit sectors.

### Data availability statement

The data that has been used is confidential.

### Declaration of interests statement

The authors declare no conflict of interest.

### Additional information

No additional information is available for this paper.

## References

[bib1] Ackerman S.J., Hilsenroth M.J. (2003). A review of therapist characteristics and techniques positively impacting the therapeutic alliance. Clin. Psychol. Rev..

[bib2] Atkinson D.R., Morten G., Sue D.W. (1979).

[bib3] American Psychological Association (2019). https://www.apa.org/workforce/data-tools/demographics.aspx.

[bib4] Beck A. (2019). Understanding Black and Minority Ethnic service user's experience of racism as part of the assessment, formulation and treatment of mental health problems in cognitive behaviour therapy. The Cognitive Behaviour Therapist.

[bib5] Bedi R.P. (2018). Racial, ethnic, cultural, and national disparities in counseling and psychotherapy outcome are inevitable but eliminating global mental health disparities with indigenous healing is not. Archiv. Sci. Psychol..

[bib6] Betancourt J.R., Green A.R., Carrillo J.E., Owusu Ananeh-Firempong I.I. (2016). Defining cultural competence: a practical framework for addressing racial/ethnic disparities in health and health care. Publ. Health Rep..

[bib7] Bps.org.uk (2017). https://www.bps.org.uk/sites/www.bps.org.uk/files/Policy/Policy%20-%20Files/BPS%20Practice%20Guidelines%20%28Third%20Edition%29.pdf.

[bib8] Braun V., Clarke V. (2006). Using thematic analysis in psychology. Qual. Res. Psychol..

[bib9] Burkard A.W., Knox S., Groen M., Perez M., Hess S.A. (2006). European American therapist self-disclosure in cross-cultural counseling. J. Counsel. Psychol..

[bib10] Cardemil E.V., Battle C.L. (2003). Guess who's coming to therapy? Getting comfortable with conversations about race and ethnicity in psychotherapy. Prof. Psychol. Res. Pract..

[bib11] Carter-Francique A., Hart A., Steward A. (2013). Black college athletes’ perceptions of academic success and the role of social support. J. Intercoll. Sport.

[bib12] Chang D.F., Berk A. (2009). Making cross-racial therapy work: a phenomenological study of clients’ experiences of cross-racial therapy. J. Counsel. Psychol..

[bib13] Chang D.F., Yoon P. (2011). Ethnic minority clients' perceptions of the significance of race in cross-racial therapy relationships. Psychother. Res..

[bib14] Christensen T.M., Brumfield K.A., J Sheperis C., Young J.S., Daniels M.H. (2010). Counseling Research: Quantitative, Qualitative, and Mixed Methods.

[bib15] Constantine M.G. (2007). Racial microaggressions against African American clients in cross-racial counseling relationships. J. Counsel. Psychol..

[bib16] Day-Vines N.L., Wood S.M., Grothaus T., Craigen L., Holman A., Dotson-Blake K., Douglass M.J. (2007). Broaching the subjects of race, ethnicity, and culture during the counseling process. J. Counsel. Dev..

[bib17] Del Re A.C., Flückiger C., Horvath A.O., Symonds D., Wampold B.E. (2012). Therapist effects in the therapeutic alliance-outcome relationship: a restricted-maximum likelihood meta-analysis. Clin. Psychol. Rev..

[bib18] Delgado D.A., Ness S., Ferguson K., Engstrom P.L., Gannon T.M., Gillett C. (2013). Cultural competence training for clinical staff: measuring the effect of a one-hour class on cultural competence. J. Transcult. Nurs..

[bib19] Drustrup D. (2020). White therapists addressing racism in psychotherapy: an ethical and clinical model for practice. Ethics Behav..

[bib20] Duff C.T., Bedi R.P. (2010). Counsellor behaviours that predict therapeutic alliance: from the client's perspective. Counsell. Psychol. Q..

[bib21] Flick U. (2018).

[bib22] Galdas P. (2017).

[bib23] Gibson C. (2020). When the river runs dry: leadership, decolonisation and healing in occupational therapy. N. Zealand J. Occupat. Ther..

[bib25] Greenberg L.S. (1986). Change process research. J. Consult. Clin. Psychol..

[bib26] Hare E.J. (2015).

[bib27] Horst K., Mendez M., Culver-Turner R., Amanor-Boadu Y., Minner B., Cook J., McCollum E. (2012). The importance of therapist/client ethnic/racial matching in couples treatment for domestic violence. Contemp. Fam. Ther..

[bib57] Knox Sarah, Burkard Alan, Johnson Adanna Jinaki, Suzuki Lisa A., Ponterotto Joseph G. (2003). African American and European American therapists’ experiences of addressing race in cross-racial psychotherapy dyads. J. Counsel. Psychol..

[bib28] Lent R.W., Lopez F.G. (2002). Cognitive ties that bind: a tripartite view of efficacy beliefs in growth-promoting relationships. J. Soc. Clin. Psychol..

[bib29] Lo H.T., Fung K.P. (2003). Culturally competent psychotherapy. Can. J. Psychiatr..

[bib31] Massao P.B., Fasting K. (2010). Race and racism: experiences of black Norwegian athletes. Int. Rev. Sociol. Sport.

[bib32] Morales K., Keum B.T., Kivlighan D.M., Hill C.E., Gelso C.J. (2018). Therapist effects due to client racial/ethnic status when examining linear growth for client- and therapist-rated working alliance and real relationship. Psychotherapy.

[bib33] Naoi A., Watson J., Deaner H., Sato M. (2011). Multicultural issues in sport psychology and consultation. Int. J. Sport Exerc. Psychol..

[bib34] Nelson D.W., Baumgarte R. (2004). Cross cultural misunderstandings reduce empathic responding. J. Appl. Soc. Psychol..

[bib35] Orlinsky D.E., Grawe K., Parks B.K. (1994).

[bib36] Owen J., Tao K.W., Imel Z.E., Wampold B.E., Rodolfa E. (2014). Addressing racial and ethnic microaggressions in therapy. Prof. Psychol. Res. Pract..

[bib37] Pedersen P., Corsini R. (1985). Innovative Psychotherapies.

[bib38] Rogers C.R. (1957). The necessary and sufficient conditions of therapeutic personality change. J. Consult. Psychol..

[bib39] Rogers-Sirin L., Melendez F., Refano C., Zegarra Y. (2015). Immigrant perceptions of therapists’ cultural competence: a qualitative investigation. Prof. Psychol. Res. Pract..

[bib40] Safran J.D. (1993). The therapeutic alliance rupture as a transtheoretical phenomenon: definitional and conceptual issues. J. Psychother. Integrat..

[bib41] Safran J.D., Crocker P., McMain S., Murray P. (1990). Therapeutic alliance rupture as a therapy event for empirical investigation. Psychother. Theor. Res. Pract. Train..

[bib42] Safran J.D., Muran J.C., Eubanks-Carter C. (2011). Repairing alliance ruptures. Psychotherapy.

[bib43] Saha S., Beach M.C., Cooper L.A. (2008). Patient centeredness, cultural competence and healthcare quality. J. Natl. Med. Assoc..

[bib44] Sigurdson K., Mitchell B., Liu J., Morton C., Gould J.B., Lee H.C., Profit J. (2019). Racial/ethnic disparities in neonatal intensive care: a systematic review. Pediatrics.

[bib45] Spiers J.A., Wood A. (2010). Building a therapeutic alliance in brief therapy: the experience of community mental health nurses. Arch. Psychiatr. Nurs..

[bib46] Sue S., Zane N., Nagayama Hall G.C., Berger L.K. (2009). The case for cultural competency in psychotherapeutic interventions. Annu. Rev. Psychol..

[bib47] Sullivan J.R. (2012). Skype: an appropriate method of data collection for qualitative interviews?. Hilltop Rev..

[bib48] Thompson V.L.S., Alexander H. (2006). Therapists' race and African American clients' reactions to therapy. Psychother. Theor. Res. Pract. Train..

[bib49] Townes D.L., Chavez-Korell S., Cunningham N.J. (2009). Re-examining the relationships between racial identity, cultural mistrust, help-seeking attitudes, and preference for a Black counselor. J. Counsel. Psychol..

[bib50] Tracy S.J. (2010). Qualitative quality: eight “big-tent” criteria for excellent qualitative research. Qual. Inq..

[bib51] Turner D. (2020).

[bib52] Vasquez M.J.T. (2007). Cultural difference and the therapeutic alliance: an evidence-based analysis. Am. Psychol..

[bib53] Wade P., Bernstein B.L. (1991). Culture sensitivity training and counselor's race: effects on Black female clients' perceptions and attrition. J. Counsel. Psychol..

[bib54] Weir K. (2018). November). A growing demand for sport psychologists. Mon. Psychol..

[bib55] West-Olatunji C.A., Frazier K.N., Guy T.L., Smith A.J., Clay L., Breaux W. (2007). The use of the racial/cultural identity development model to understand a Vietnamese American: a research case study. J. Multicult. Counsel. Dev..

[bib56] Yeo E., Torres-Harding S.R. (2021). Rupture resolution strategies and the impact of rupture on the working alliance after racial microaggressions in therapy. Psychotherapy.

